# Supplementary material for: Chagas Disease-Related Mortality in Spain, 1997 to 2018
Source: Microorganisms. 2021 Sep 20;9(9):1991. doi: 10.3390/microorganisms9091991 (PMC8469044; doi:10.3390/microorganisms9091991)
Supplement: Supplementary file 1 [file microorganisms-09-01991-s001.zip › microorganisms-1358245-supplementary.pdf]

Table S1. Included codes of the International Classification of Diseases-Ninth Revision, Clinical Modification (ICD-9-CM) and ICD-10-CM

**Chagas diseases**	**ICD-9-CM**	**ICD-10-CM**
Trypanosomiasis	086	
Chagas disease of heart	086.0	
Chagas disease of other organ	086.1	
Chagas disease unspecified	086.2	
Trypanosomiasis unspecified	086.9	
Chagas disease		B57
Chagas disease acute with involvement of heart		B57.0
Acute Chagas disease with heart implications		B57.1
Chronic hearth diseases with heart implications		B57.2
Chronic Chagas disease with implication of digestive tract		B57.3
Chronic Chagas disease with involvement of central nervous system		B57.4
Chronic Chagas disease with other organism affected		B57.5
**Comorbidities**		
Diabetes mellitus	250	E08 – E13
Heart failure	428	I50
Ischemic cardiopathy	410-414	I20 – I25
Cerebrovascular diseases	430-438	I60 – I69
Chronic lung diseases	490 – 496	J40 – J47
Chronic kidney failure	585	N18
Neoplasms	140-239	C00 – C96
Lymphoma/leukemia	200 - 208	C81 – C96
Human immunodeficiency virus (HIV)	042, 079.53	B20

Table S2. Case fatality rate (CFR) in hospitalized patients in Spain with and without Chagas disease, by age

Age	Chagas disease			Non-Chagas disease			Difference (95% CI)*
	N deaths / N cases	% of total deaths	CFR	N deaths / N cases	% of total deaths	CFR	
0-9 years	2/149	3.6	1.3	59,421/ 6371,554	1.9	0.9	0.4 (−0.5, 3.8)
10-19 years	1/50	1.8	2.0	9368/ 5,711,142	0.3	0.2	1.8 (0.9, 10.3)
20-29 years	2/699	3.6	0.3	19,816/ 6,652,099	0.6	0.3	0.0 (−0.8,0.2)
30-39 years	7/1888	12.5	0.4	44,517/ 9,977,510	1.4	0.4	0.0 (−0.3, 0.2)
40-49 years	8/1086	14.3	0.7	112,136/ 6,865,029	3.5	1.6	−0.9 (−1.9, −1.3)
50-59 years	12/684	21.4	1.8	237,046/ 8,236,456	7.5	2.9	−1.1 (−1.8, −0.1)
60-69 years	11/340	19.6	3.2	441,989/ 10,876,641	13.9	4.1	−0.8 (−1.6, −0.2)
70-79 years	7/89	12.5	7.9	835,545/ 13,713,748	26.4	6.1	1.8 (−2.2, 9.2)
80-89 years	5/30	8.9	16.7	1,067,728 / 10,171,384	33.7	10.5	6.1 (−3.1, 23)
90-100 years	1/7	1.8	14.3	371,125/ 2,137,996	11.7	17.4	−3.1 (−33, 14)
Total	56/5022	1.1	100	3,170,961 / 77,729,072	100	4.1	−2.9 (−2.6, −3.2)

*Differences: CFR for Chagas patients minus CFR for non-Chagas patients

Table S3. Characteristics of patients whose deaths were directly related to Chagas disease

N	Age /Sex	Year	Country of birth	Diagnoses (main diagnosis in bold)[a]	Length of admission, days
1	60/F	2007	NA	**Cardiac arrest**<br>Chagas disease with heart involvement<br>Diseases of the tricuspid valve<br>Other non-infectious gastroenteritis/colitis<br>Nephrotic syndrome with proliferative glomerulonephritis lesion	0
2	44/F	2007	NA	**Chagas disease with heart involvement**<br>Unspecified non-psychotic mental disorder as a result of organic brain damage<br>Other primary myocardiopathies<br>Unspecified cardiac dysrhythmia<br>Left heart failure<br>Acute pancreatitis<br>Shock without mention of trauma (other)<br>Epigastric abdominal pain<br>Automatic implanted cardiac defibrillator	3
3	61/M	2008	NA	**Primary cardiomyopathy (others)**<br>Chagas disease without organ involvement<br>Unspecified heart failure<br>Unspecified acute renal failure	8
4	52/M	2009	NA	**Chagas disease with heart involvement**<br>Mitral valve disorders<br>Tricuspid valve disorders specified as non-rheumatic<br>Phlebitis and thrombophlebitis from other sites (others)<br>Unspecified pleural effusion<br>Cardiogenic shock<br>Ascites<br>Bacteremia<br>Infectious and inflammatory reaction by devices implants and vascular grafts<br>Hematoma that complicates a procedure.	38
5	31/M	2009	NA	**Chagas disease with involvement of another organ**<br>Clostridium difficile infection<br>HIV<br>Cytomegalovirus disease<br>Tobacco use disorder<br>Hemiplegia (affectation of side not specified)<br>Epilepsy (no mention of untreatable epilepsy)<br>Pneumonitis due to inhalation of food or vomit<br>Urinary tract infection (site not specified)<br>Infectious and parasitic diseases (others)<br>Other urinary problems	65
6	44/F	2010	NA	**Chagas disease with heart involvement**<br>Right bundle blanch block<br>Complications of transplanted heart<br>Colon diverticulosis with no mention of hemorrhage<br>Chagas disease with involvement of another organ<br>Other specified intestinal disorders (other)<br>Diaphragmatic hernia<br>Unspecified myalgia and myositis<br>Post-operative shock<br>Cardiogenic shock	2

N	Age /Sex	Year	Country of birth	Diagnoses (main diagnosis in bold)[a]	Length of admission, days
				SIRS by a non-infectious process with acute organic dysfunction<br>Unspecified acute renal failure<br>Automatic implanted cardiac defibrillator<br>Organ transplant waiting status.	
7	65/F	2011	NA	**Chagas disease with heart involvement**<br>Other primary myocardiopathies<br>Unspecified congestive heart failure<br>Cardiogenic shock<br>Other unspecified coagulation defects<br>Acidosis<br>Other abnormal glucose<br>Unspecified kidney failure (Unspecified Kidney Failure)<br>Automatic implanted cardiac defibrillator	14
8	34/F	2012	NA	**Cardiogenic shock**<br>Chagas disease without organ involvement<br>Idiopathic myocarditis<br>Other primary myocardiopathies<br>Pericardial disease not specified.	0
9	64/F	2012	NA	**Unspecified congestive heart failure**<br>Acute over chronic systolic heart failure<br>Chagas disease with heart involvement<br>Other primary myocardiopathies<br>Unspecified acute renal failure<br>Oliguria and anuria<br>Atrial fibrillation<br>Mitral valve insufficiency and aortic valve insufficiency<br>Diseases of the tricuspid valve<br>Chronic cardiopulmonary disease (others)<br>Unspecified hypothyroidism<br>Stage II chronic nephropathy (mild)<br>Obesity not specified<br>Cardiac pacemaker	2
10	47/F	2012	NA	**Chagas disease with involvement of another organ**<br>Bleeding that complicates a procedure<br>Dysfunction of the sinoatrial node<br>Cardiac pacemaker<br>Prolonged (current) use of anticoagulants	34
11	55/F	2013	NA	**Chagas disease with involvement of another organ**<br>Cardiac arrest<br>Ventricular fibrillation<br>Hypokalemia<br>Cardiomyopathy in other diseases classified under other concepts<br>Unspecified essential hypertension<br>Left heart failure<br>Atrial fibrillation<br>Esophageal reflux<br>Another blocking of the left branch of the booklet<br>Transient ischemic attack (TIA) and cerebral infarction without residual deficit<br>Prolonged (current) use of anticoagulants	15
12	75/F	2013	NA	**Chagas disease with heart involvement**<br>Other primary myocardiopathies<br>Unspecified congestive heart failure	27

N	Age /Sex	Year	Country of birth	Diagnoses (main diagnosis in bold)[a]	Length of admission, days
				Acute purulent endophthalmitis<br>Hypoosmolality and/or hyponatremia<br>Stage I to stage IV chronic hypertensive kidney disease (unspecified)<br>Chronic nephropathy, not otherwise specified<br>Cardiac pacemaker<br>Prolonged (current) use of anticoagulants	
13	60/M	2013	NA	**Congestive heart failure, unspecified**<br>Acute respiratory failure<br>Chagas disease with heart involvement<br>Automatic implanted cardiac defibrillator<br>Prolonged (current) use of anticoagulants<br>Obesity not specified<br>Unspecified hearing loss<br>Pterygium not specified	16
14	43/F	2013	NA	**Chagas disease with heart involvement**<br>Cardiac arrest<br>Anoxic brain damage	6
15	77/M	2016	Peru	**Chagas disease (chronic) affecting the heart**<br>Ventricular tachycardia<br>Atherosclerotic heart disease of native coronary artery without angina<br>Unspecified left ventricular failure<br>Accidental puncture and tearing of an organ or structure of the circulatory system during a circulatory system procedure<br>Cardiac catheterization as a cause of abnormal patient reaction or subsequent complication with no mention of adverse event at the time of the procedure<br>Post-procedure (acute) (chronic) renal failure	17
16	64	2016	Colombia	**Cardiac arrest, cause not specified**<br>Chagas disease (chronic) affecting the heart<br>Non-toxic multinodular goiter<br>Hyperlipidemia not specified<br>Epileptic syndrome with absences (not untreatable) without status epilepticus<br>Unspecified encephalopathy<br>Essential (primary) hypertension<br>Paroxysmal atrial fibrillation<br>Unspecified heart failure<br>Venous insufficiency (chronic) (peripheral)<br>Pneumonia (microorganism not specified)<br>Cardiac achalasia<br>Colon diverticulosis without perforation or abscess (no bleeding)<br>Erythema nodosum<br>Primary osteoarthritis of the knee bilateral<br>Spondylosis not specified<br>Stage 5 chronic kidney disease<br>Prolonged (current) use of anticoagulants	11
17	56	2016	NA	**Chagas disease (chronic) affecting the heart**<br>Cardiomyopathy in diseases classified under another concept<br>Cardiogenic shock<br>Systemic inflammatory response syndrome (SIRS) of non-infectious origin with acute organic dysfunction<br>Unspecified acute renal failure<br>Acute and subacute liver failure without coma	49

N	Age /Sex	Year	Country of birth	Diagnoses (main diagnosis in bold)[a]	Length of admission, days
				Disseminated intravascular coagulation (defibrillation syndrome)	
				Diabetes mellitus type 2 without complications	
				Primary essential hypertension	
18	51	2018	Bolivia	**Chagas disease with cardiac involvement**	108
				Megacolon in Chagas disease	
				Mucocutaneous leishmaniasis	
				Blood group A (Rh positive)	
				Bleeding from anus and rectum	
				Other hemorrhoids	
				Acute post-hemorrhagic anemia	
				Personal history of other infectious and parasitic diseases	
				Uncomplicated alcohol dependency	
				Occupational exposure to toxic agents in other industries	
				Cushing's syndrome not specified	
				Lumbago with sciatica (side not specified)	
				Hypokalemia	
				Hypoosmolality and hyponatremia	
				Constipation not specified	
				Unspecified caloric-protein malnutrition	
				Muscle loss and atrophy not otherwise classified (location not specified)	
				Adverse effect of antifungal antibiotics used by systemic route (initial contact)	
				Immunodeficiency not specified	
				Personal history of immunosuppression therapy	
19	51	2018	Bolivia	**Chagas disease (chronic) affecting the heart**	3
				Contact for palliative care	
				Other type of urinary retention	
				Unspecified heart failure	
				Presence of cardiac pacemakers	
				Atrial fibrillation not specified	
				Prolonged (current) use of anticoagulants	
20	63	2018	Spain	**Chagas disease with digestive system involvement, unspecified**	4
				Chagas disease (chronic) affecting the heart	
				Unspecified caloric-protein malnutrition	
				Muscle loss and atrophy not otherwise classified (location not specified)	
				Poorly defined and unknown causes of mortality	
				Primary biliary cirrhosis	
				Polyneuropathies in diseases classified under another concept	
				Barrett's esophagus without dysplasia	
				Oropharyngeal phase dysphagia	
				Autoimmune hepatitis	
				Fatty liver (fat degeneration) not otherwise classified	
				Acquired absence of another (-s) genital organ (-s)	
				Acquired absence of both the cervix and the uterus	

M: male, F: female, NA: not available; SIRS: Systemic inflammatory response syndrome

[a] The order of secondary diagnosis is the same as recorded in the original database

Table S4. Characteristics of included patients whose deaths were not directly attributable to Chagas disease

N	Age/ Sex	Year	Country	Diagnoses (main diagnosis in bold)[a]	Length of admission, days
1	31/ M	2002	NA	**Unspecified viral hepatitis without liver coma**<br>Chagas disease without organ involvement<br>Other primary myocardiopathies<br>First degree atrioventricular block<br>Cardiac pacemaker	18
2	82/F	2002	NA	**Bowel obstruction (other)**<br>Chagas disease without organ involvement<br>Unspecified protein-calorie malnutrition<br>Cachexia	12
3	83/ M	2005	NA	**Pulmonary embolism and heart attack (other)**<br>Chagas disease with heart involvement<br>Blocking of the right branch of the booklet<br>Atrial fibrillation<br>Unspecified location ruptured aortic aneurysm<br>Cardiogenic shock	0
4	27/ M	2005	NA	**Acute myeloid leukemia with no mention of having reached remission**<br>Chagas disease with involvement of another organ<br>Trypanosomiasis not specified<br>Candidiasis enteritis<br>Encephalitis myelitis and encephalomyelitis in protozoan diseases classified under other concepts<br>Irritable colon<br>Unspecified acute renal failure<br>Diarrhea<br>Hepatomegaly<br>Infectious and parasitic diseases (others)	49
5	41/ M	2007	NA	**HIV disease**<br>Septicemia not specified<br>E coli infection<br>Chagas disease without organ involvement<br>Thrush<br>Meningoencephalitis due to toxoplasmosis<br>Volume decrease<br>Unspecified non-infectious gastroenteritis/colitis<br>Septic shock<br>Severe sepsis	22
6	31/F	2007	NA	**Pneumonia**<br>Mycoplasma infection<br>Chagas disease without organ involvement<br>Other unspecified coagulation defects<br>Unspecified acute pericarditis<br>Acute and subacute liver necrosis<br>Unspecified kidney failure (Unspecified Kidney Failure)<br>Septic shock<br>Non-specific elevation of transaminase or lactodeshydrogenase (LDH) levels	7
7	0/M	2007	NA	**Hypoplastic left heart syndrome**<br>Chagas disease without organ involvement	5

N	Age/ Sex	Year	Country	Diagnoses (main diagnosis in bold)[a]	Length of admission, days
				Maternal infections	
8	58/F	2008	NA	**Benign neoplasm of the brain**<br>Chagas disease without organ involvement<br>Dysphagia	12
9	65/F	2009	NA	**Unspecified cerebral artery occlusion with cerebral infarction**<br>Other staphylococcal sepsis<br>Chagas disease without organ involvement<br>Diabetes mellitus type II without mention of complication<br>Hyperkaliemia<br>Quadriplegia and quadriparesis (others)<br>Status of great evil<br>Unspecified essential hypertension<br>Cardiomyopathy in other diseases classified under other concepts<br>Right bundle branch block and left anterior fascicular block<br>Atrial fibrillation<br>Coma<br>Sepsis	9
10	26/F	2009	NA	**Gallbladder stone with acute cholecystitis no mention of obstruction**<br>HIV disease<br>Cytomegalovirus disease<br>Chagas disease without organ involvement<br>Anemia not specified<br>Other unspecified coagulation defects<br>Hemoperitoneum (non-traumatic)<br>Liver abscess<br>Other specified liver disorders<br>Other specified disorders of the biliary tract<br>Complications of the digestive system<br>Other interventions and surgical techniques specified<br>Personal history of allergy to another antibiotic agent	168
11	32/F	2010	NA	**Cerebral edema**<br>Congenital factor VIII disorder<br>Non-pyogenic meningitis<br>Chagas disease without organ involvement<br>Personal history of tuberculosis	2
12	11/F	2011	NA	**Intracerebral hemorrhage**<br>Other seizures<br>Meningitis not specified<br>Chagas disease without organ involvement<br>Other complications by device implant and graft of the nervous system<br>Surgical intervention with implantation of an artificial internal device<br>Other states after a procedure (other)	46
13	54/F	2011	NA	**Malignant heart neoplasm**<br>Secondary malignant neoplasm of the pleura<br>Secondary malignant neoplasm from other specified sites (other)<br>Pain (acute/chronic) related to a neoplasm<br>Unspecified acute pericarditis<br>Chagas disease with heart involvement<br>Sicca syndrome<br>Mitral valve disorders<br>Unspecified hypothyroidism<br>Chronic lymphocytic thyroiditis	9

N	Age/ Sex	Year	Country	Diagnoses (main diagnosis in bold)[a]	Length of admission, days
14	54/ M	2011	NA	**Bronchopneumonia**<br>Septicemia not specified<br>Severe sepsis<br>Septic shock<br>Unspecified acute renal failure<br>Other symptoms that affect the nervous system and the musculoskeletal system<br>Acute respiratory failure<br>Other sequelae of chronic liver disease<br>Shock not specified<br>Recurrent acute lymphoid leukemia<br>Chagas disease without organ involvement<br>Unspecified essential hypertension<br>Neutropenia not specified	8
15	88/ M	2012	NA	**Visceral herpes simplex**<br>Unspecific esophagitis<br>Unspecified gastrointestinal tract bleeding<br>Shock without mention of trauma (other)<br>Chagas disease without organ involvement<br>Ancient myocardial infarction<br>Percutaneous transluminal coronary angioplasty<br>Blood vessel replaced by other means<br>Unspecified hypothyroidism<br>Colostomy<br>Pneumonitis due to inhalation of food or vomit	11
16	50/ M	2012	NA	**Toxoplasmosis from other specified sites**<br>Brain compression<br>Chagas disease without organ involvement<br>Unspecified bowel obstruction<br>Pneumonia	32
17	39/F	2013	NA	**Pneumonia**<br>Acute respiratory failure<br>HIV<br>Septicemia<br>Trypanosomiasis not specified<br>Cocaine dependence<br>Protein-calorie malnutrition<br>Cachexia<br>Anemia	1
18	4/F	2013	NA	**Fever unspecified**<br>Infantile cerebral palsy not specified<br>Thrombocytopenia not specified<br>Hematemesis<br>Unspecified protein calorie malnutrition<br>Unspecified acute renal failure<br>Dehydration<br>Chagas disease without organ involvement<br>Generalized convulsive epilepsy (no mention of untreatable epilepsy)<br>Open wound of the foot (except toes only) without mention of complication<br>Residential institution	14
19	71/ M	2014	NA	**Malignant neoplasm of the brain (other parts of the brain)**<br>Cellulite/abscess in trunk	12

N	Age/ Sex	Year	Country	Diagnoses (main diagnosis in bold)[a]	Length of admission, days
				Untreated epilepsy not specified<br>Late syphilis not specified<br>Chagas disease without organ involvement<br>Hypertensive heart disease without heart failure<br>Chronic bronchitis not specified<br>Uncomplicated secondary diabetes mellitus<br>Adrenal cortex steroids<br>History of tobacco use<br>Other diseases of the respiratory system not classified under other concepts	
20	41/ M	2014	NA	**Influenza with pneumonia**<br>Other pulmonary insufficiencies not otherwise classified<br>Bronchitis not specified as acute or chronic<br>Pseudomonas infection<br>Septicemia by other gram-negative organisms (others)<br>Sepsis<br>Urinary tract infection (site not specified)<br>Klebsiella pneumoniae infection<br>Other tracheostomy complications<br>Other specified cardiac dysrhythmias (other)<br>Chagas disease without organ involvement<br>Diabetes mellitus type II without mention of complication	22
21	58/ M	2014	NA	**Other hand and wrist tenosynovitis**<br>Non-traumatic upper limb compartment syndrome<br>Upper extremity arterial embolism and thrombosis<br>Acute post-hemorrhagic anemia<br>Fever present in diseases classified under other concepts<br>Atrial fibrillation<br>Rhabdomyolysis<br>Chagas disease with involvement of another organ<br>Another sharp pain<br>Unspecified multi-site arthropathy<br>Septicemia not specified<br>Severe sepsis<br>History of tobacco use<br>Prolonged (current) steroid use	24
22	79/ M	2016	Bolivia	**Other types of bowel obstruction**<br>Megacolon in Chagas disease<br>Other specified congenital malformations of the intestine<br>Diabetes mellitus type 2 without complications<br>Essential (primary) hypertension<br>Unspecified interstitial lung disease<br>Bronchiectasis without complications<br>Unspecified Alzheimer's disease<br>Dementia in other diseases classified under another concept (without behavioral alteration)<br>Personal history of tuberculosis<br>Personal history of pulmonary embolism	5
23	40/F	2016	Bolivia	**Rectosigmoid junction malignancy**<br>Secondary malignant neoplasm of the liver and intrahepatic bile ducts<br>Secondary malignant neoplasm of retroperitoneum and peritoneum<br>Chagas disease (chronic) affecting the heart<br>Unspecified hypothyroidism	9

N	Age/ Sex	Year	Country	Diagnoses (main diagnosis in bold)[a]	Length of admission, days
				Problems related to living alone<br>Colostomy status	
24	65/ M	2016	Bolivia	**Bleeding esophageal ulcer**<br>Pemphigus erythematosus<br>Stage 5 chronic kidney disease<br>Renal dialysis dependency<br>Diabetes mellitus type 2 without complications<br>Prolonged (current) use of insulin<br>Secondary hyperparathyroidism of renal origin<br>Colon diverticulosis without perforation or abscess (no bleeding)<br>Diaphragmatic hernia without obstruction or gangrene<br>Benign prostatic hyperplasia without lower urinary tract symptoms<br>Acute Chagas disease that does not affect the heart	33
25	43/ M	2016	NA	**Agranulocytosis secondary to antineoplastic chemotherapy**<br>Hypotension not specified<br>Other complications of bone marrow transplantation<br>Graft-versus-host disease not specified<br>Unspecified erythematous condition<br>Unspecified cytomegalovirus disease<br>Other leukemias specified in recurrence<br>Chagas disease that does not affect the heart<br>Personal history of antineoplastic chemotherapy<br>Personal history of nicotine dependence	8
26	70/ M	2017	United Kingdom	**Nutritional marasmus**<br>Stupor<br>Stage IV sacral pressure ulcer<br>Infection with methicillin-resistant Staphylococcus aureus as a cause of diseases classified under another concept<br>Stage IV left buttock pressure ulcer<br>Left hip pressure ulcer stage III<br>Stage III right ankle pressure ulcer<br>Neglect or abandonment of adults (confirmed) initial contact<br>Acute Chagas disease with cardiac involvement<br>Unspecified iron deficiency anemia<br>Unspecified dementia without altered behavior<br>State of allergy to penicillin	51
27	90/ M	2017	NA	**Mane**<br>Hematemesis<br>Liver failure (not specified) without a coma<br>Thrombosis of the portal vein<br>Liver cell carcinoma<br>Chagas disease (chronic) affecting the heart<br>Essential (primary) hypertension<br>Diabetes mellitus type 2 without complications<br>Portal hypertension<br>Acute post-hemorrhagic anemia<br>Stage II left buttock pressure ulcer<br>Stage I right heel pressure ulcer<br>Stage I left heel pressure ulcer	1

N	Age/ Sex	Year	Country	Diagnoses (main diagnosis in bold)[a]	Length of admission, days
28	76/ M	2017	Spain	**Cerebral infarction due to unspecified occlusion or stenosis of the left middle cerebral artery**<br>Benign intracranial hypertension<br>Pneumonia due to unspecified microorganisms<br>Viral Hepatitis B unspecified without a hepatic coma<br>Chagas disease (chronic) affecting the heart<br>Chronic obstructive pulmonary disease with exacerbation (acute)<br>Essential (primary) hypertension<br>Nicotine (cigarette) dependence without complications<br>Ancient myocardial infarction<br>Presence of angioplasty grafts and coronary prosthesis<br>Prolonged (current) use of aspirin<br>State of narcotic allergy	3
29	51/ M	2017	Bolivia	**Malignant neoplasm of the brain except lobes and ventricles**<br>Sepsis (microorganism not specified)<br>Urinary tract infection (location not specified)<br>Contact for palliative care<br>Other specified respiratory disorders<br>Other specified post-procedure states<br>Personal history of nicotine dependence<br>Chagas disease (chronic) affecting the heart	20
30	69/F	2017	Brazil	**Acute myocardial infarction with ST elevation of unspecified location**<br>Essential (primary) hypertension<br>Hyperlipidemia not specified<br>Unspecified cardiomyopathy<br>Presence of automatic (implantable) cardiac defibrillator<br>Persistent atrial fibrillation<br>Cardiogenic shock<br>Ventricular fibrillation<br>Chagas disease (chronic) affecting the heart<br>Other specified non-inflammatory vaginal disorders	4
31	86/F	2018	Spain	**Acute bronchitis unspecified**<br>Handset flutter not specified<br>Chronic atrial fibrillation<br>Unspecified iron deficiency anemia<br>Rheumatic mitral stenosis with insufficiency<br>Chagas disease (chronic) affecting the heart<br>Personal history of pulmonary embolism<br>Colon diverticulosis without perforation or abscess (no bleeding)<br>Diaphragmatic hernia without obstruction or gangrene<br>Unspecified hypothyroidism<br>Widespread primary osteoarthritis<br>Mild cognitive impairment<br>Wheelchair dependency	3
32	85/ M	2018	Bolivia	**Pneumonitis due to food inhalation and vomiting**<br>Acute respiratory failure not specified if with hypoxia or hypercapnia<br>Chagas disease (chronic) affecting the heart<br>Unspecified Alzheimer's disease<br>Dementia in other diseases classified under another concept without altering behavior<br>Epilepsy and other generalized (non-intractable) epileptic syndromes without status epilepticus<br>Hyperlipidemia not specified	6

N	Age/ Sex	Year	Country	Diagnoses (main diagnosis in bold)[a]	Length of admission, days
				Nicotine (cigarette) dependence without complications<br>Prolonged (current) use of aspirin	
33	52/ M	2018	Bolivia	**Rectosigmoid junction malignancy**<br>Secondary malignant neoplasm of retroperitoneum and peritoneum<br>Unspecified inflammatory prostate disease<br>Erectile dysfunction due to other diseases classified under another concept<br>Personal history of nicotine dependence<br>Alcohol dependency in remission<br>Cocaine dependence in remission<br>Chagas disease with unspecified digestive system involvement<br>Unspecified esophagitis<br>Diaphragmatic hernia without obstruction or gangrene<br>Personal history of other infectious and parasitic diseases	45
34	62/F	2018	Bolivia	**Malignant neoplasm of the ascending colon**<br>Other specified functional bowel disorders<br>Adverse effect of unspecified narcotics (initial contact)<br>Pain (acute) (chronic) related to neoplasia<br>Secondary malignant neoplasm of retroperitoneum and peritoneum<br>Secondary malignant neoplasm of the liver and intrahepatic bile ducts<br>Contact for palliative care<br>Cachexia<br>First degree hemorrhoids<br>Unspecified chronic gastritis without bleeding<br>Chagas disease (chronic) affecting the heart	5
35	36/F	2018	Uruguay	**Agranulocytosis secondary to antineoplastic chemotherapy**<br>Adverse effect of antineoplastic and immunosuppressive drugs (initial contact)<br>Thrombocytopenia not specified<br>Unspecified oral mucositis (ulcer)<br>Unspecified acute renal failure<br>Malignant neoplasm of the rectum<br>Secondary malignant neoplasm of the brain<br>Secondary malignant neoplasm of retroperitoneum and peritoneum<br>Secondary malignant neoplasm of the right lung<br>Secondary malignant neoplasm of the left lung<br>Secondary malignant neoplasm of the liver and intrahepatic bile ducts<br>Secondary malignant neoplasm of the right adrenal gland<br>Chagas disease (chronic) affecting other organs	3
36	73/ M	2018	Ecuador	**Sepsis by gram negatives no specified**<br>Severe sepsis with septic shock<br>Unspecified infectious gastroenteritis and colitis<br>Unspecified acute renal failure<br>Liver failure (not specified) without a coma<br>Acidosis<br>Unspecified coagulation defect<br>Hypertensive chronic kidney disease with stage 1 to 4 chronic kidney disease or unspecified chronic kidney disease<br>Stage 3 chronic kidney disease (moderate)<br>Diabetes mellitus type 2 without complications<br>Hyperlipidemia not specified<br>Obesity not specified<br>Gallbladder stone without cholecystitis without obstruction	1

N	Age/ Sex	Year	Country	Diagnoses (main diagnosis in bold)[a]	Length of admission, days
				Dilated cardiomyopathy	
				Chagas disease (chronic) affecting the heart	
				Non-rheumatic mitral (valve) insufficiency	
				Non-rheumatic tricuspid (valve) insufficiency	
				Chronic atrial fibrillation	
				Presence of cardiac pacemakers	
				Prolonged (current) use of anticoagulants	

F: female M: male; NA: not available.

[a] The order of secondary diagnosis is the same as recorded in an original database
